# A descriptive content analysis of greenwashing tactics used in US cigarette advertisements between 2019–2023

**DOI:** 10.18332/tpc/213722

**Published:** 2026-04-30

**Authors:** Maryam Ibrahim, Meghan B. Moran, Lauren Czaplicki, Ryan D. Kennedy, Dana Tfayli, Gideon P. Naudé, Justin C. Strickland, Matthew W. Johnson

**Affiliations:** 1 Department of Health, Behavior and Society, Johns Hopkins Bloomberg School of Public Health, Baltimore, United States; 2 Department of Psychiatry and Behavioral Sciences, Johns Hopkins University School of Medicine, Baltimore, United States

**Keywords:** tobacco control, tobacco industry, cigarettes, advertising, smoking prevention

## Abstract

**INTRODUCTION:**

The tobacco industry has incorporated strategies like greenwashing (i.e. a marketing tactic that utilizes false or unverified claims to mislead consumers about a business's environmental practices and impact) within cigarette advertisements. Despite regulation to limit greenwashing, research found continued employment of greenwashing tactics. Understanding the magnitude and extent of greenwashing strategies used by the industry is helpful given the emergence of these alternative tactics and the association between greenwashing advertising and consumer inaccurate risk perceptions.

**METHODS:**

We conducted a descriptive content analysis of 2102 cigarette ads that ran January 2019–December 2023 in the US and identified 487 ads (23.2%) that had at least one greenwashing feature. We further characterized the nature of the greenwashing tactics present via text, imagery, or audio cues in the ads, using a developed codebook. Ads were independently double-coded, with discrepancies reconciled by team deliberation.

**RESULTS:**

Over 90% of the sample of ads came from 4 brands: Hestia, Natural American Spirit, Winston and Marlboro. Social media were predominant for ad identification. Hestia ads predominantly featured the descriptors ‘naked’ (74.6%) and ‘wild’ (63.9%), and flora imagery (67.3%). Natural American Spirit ads frequently used descriptors such as ‘different’ (50.9%) and ‘simple’ (41.1%), and over half (58.9%) featured flora imagery. Winston ads used the term ‘tobacco and water’ (45.0%) and depicted the great outdoors (47.0%). Marlboro ads commonly used great outdoors imagery (92.7%) and eco-related sweepstakes (41.5%).

**CONCLUSIONS:**

Greenwashing continues to be used in cigarette advertising, including the use of tactics associated with inaccurate modified risk perceptions. If further studies strengthen the evidence, regulations to limit greenwashing in tobacco advertising may be justified.

## INTRODUCTION

For years, the US tobacco industry has used deceptive marketing tactics to mislead consumers into believing their products are less harmful than in reality^1^. Some of these tactics, such as use of low-tar, light, and mild descriptors, have been restricted^2^. However, to circumvent these restrictions, the industry has incorporated alternative strategies like greenwashing (i.e. a marketing tactic that utilizes false or unverified claims to mislead consumers about a business’s environmental practices and impact^3^)^4-8^. The use of greenwashing content within cigarette advertisements is associated with inaccurate consumer harm and addictiveness perceptions^9-13^. Greenwashing also has the potential to convey that one cigarette product is safer than another^14^.

Given the association between greenwashed advertising and inaccurate reduced risk perceptions, the U.S. Food and Drug Administration (FDA) took action to limit one specific greenwashing strategy – the use of ‘additive-free’ and/or ‘natural’ – by the manufacturers of Natural American Spirit (NAS) cigarettes and Nat Sherman cigarettes^15^. Despite this, research found that both companies continued to employ a variety of other greenwashing tactics including use of terms such as ‘earth-friendly’, ‘simple’, ‘tobacco and water’ and imagery such as leaves, flora, nature and farm settings within the advertisement of their products^6,16,17^.

Understanding the magnitude and extent of greenwashing strategies used by the tobacco industry is helpful given the emergence of these alternative tactics and the association between greenwashing advertising and consumer inaccurate risk perceptions^11^. As these emergent tactics have the potential to mislead consumers, capturing their prevalence in extant tobacco advertising can lead to better systematic evaluation and regulation of their use. The current study describes the presence of greenwashed text and imagery within cigarette ads from 2019 through 2023.

## METHODS

### Sample

We conducted a descriptive content analysis of 2102 cigarette ads that ran January 2019–December 2023 in the US and identified a final sample of 487 ads (23.2%) that had at least one greenwashing feature. Ads were obtained from MediaRadar (a market research firm that surveils media and captures advertisements, previously two separate research firms known as Kantar Media and Numerator; n=99)^18^, the Trinkets and Trash database of tobacco ads hosted by Rutgers Institute for Nicotine and Tobacco Studies (n=151)^19^ and by downloading social media posts from official cigarette brand accounts (n=237). The sample contained a combination of audio, print, video, and visual ads.

### Coding procedures

Ads were first independently evaluated by two coders for presence of any greenwashing, defined as the use of text, imagery, or audio cues to associate the brand or product with naturalness, sustainability, simple ingredient lists, eco-friendliness, or other pro-environment/pro-nature issues. We identified our final sample of 487 ads that had any greenwashing features.

Next, we further characterized the nature of the greenwashing tactics present in the 487 ads, using a codebook (Supplementary file Table 1) developed based on existing research^6,7,16,20^ and on an initial review of advertisements. Codes captured: 1) specific descriptive keywords/phrases or close variations (e.g. natural, naturally; organic, organically); 2) general, broader textual claims about eco-friendly practice or values (e.g. that the company is committed to sustainability); 3) presence of greenwashed imagery (e.g. farms, plants); and 4) promotions connected to greenwashing (e.g. giveaway of a packet of seeds). Codes were not mutually exclusive; one ad could contain multiple codes. Coders were trained on the codebook to a reliability standard >0.8 and were not allowed to commence coding until this standard was achieved. All ads were independently coded by two-coders, and discrepancies were reconciled by study team deliberation when needed.

### Analysis

Analyses were conducted using Stata 15^21^. Inter-coder reliability was assessed using Krippendorf’s alpha (prevalence-adjusted bias-adjusted kappa statistic was used for rare codes)^22^ to ensure reliability met or exceeded a threshold of 0.8. We first produced descriptive statistics to characterize the sample by brand, channel and presence of each greenwashing tactic by number of observations and percentages of the full sample. We then ran bivariate analyses of the presence of each tactic by brand and by year.

## RESULTS

Of all 2102 cigarette ads reviewed, 23.2% contained at least one greenwashing feature, resulting in a final sample of 487 ads. The remaining 1615 ads did not have any greenwashing features and were excluded for our final sample. Table 1 presents the brand and channel characteristics. Of the 12 brands in our sample, over 90% of ads came from 4 brands: Hestia (n=205; 42.1% of the sample), Natural American Spirit (n=112; 23.0%), Winston (n=100; 20.5%) and Marlboro (n=41; 8.4%). Social media were predominant for ad identification (n=240; 49.3% of the sample), followed by consumer magazines (n=101; 20.7%) and opt-in emails (n=99; 20.3%).

**Table 1:** Descriptives (by number of observations, n, and percentages) of a content analysis of 487 US cigarette advertisements by brand and medium from 2019–2023

	*2019* *(n=101)*		*2020* *(n=61)*		*2021* *(n=34)*		*2022* *(n=152)*		*2023* *(n=139)*		*Total* *(n=487)*	
	*n*	*%*	*n*	*%*	*n*	*%*	*n*	*%*	*n*	*%*	*n*	*%*
**Brand name**												
Hestia	0	0.0	0	0.0	0	0.0	105	69.1	100	71.9	205	42.1
Natural American Spirit (NAS)	36	35.6	24	39.3	26	76.5	19	12.5	7	5.0	112	23.0
Winston	9	8.9	33	54.1	6	17.7	26	17.1	26	18.7	100	20.5
Marlboro	35	34.7	1	1.6	1	2.9	1	0.7	3	2.2	41	8.4
Nat Sherman	12	11.9	0	0.0	0	0.0	0	0.0	0	0.0	12	2.5
Aura	4	4.0	3	4.9	0	0.0	0	0.0	0	0.0	7	1.4
Other* [Parliament, Camel, Leaf by Lane, Lucky Strike, Signal, Very Low Nicotine (VLN), etc.]	5	5.0	0	0.0	1	2.9	1	0.7	3	2.2	10	2.1
**Medium**												
Social media	0	0.0	7	11.5	2	5.9	105	69.1	126	90.7	240	49.3
Consumer magazine	14	13.9	16	26.2	22	64.7	36	23.7	13	9.4	101	20.7
Opt-in Email	65	64.4	17	27.9	7	20.6	10	6.6	0	0.0	99	20.3
Direct-to-consumer (DTC) mail	19	18.8	6	9.8	2	5.9	0	0.0	0	0.0	27	5.5
Online	3	3.0	15	24.6	0	0.0	0	0.0	0	0.0	18	3.7
Outdoor	0	0.0	0	0.0	1	2.9	1	0.7	0	0.0	2	0.4

* One ad contained 8 Premier Manufacturing cigarette brands advertised [Ace, Manitou, Shield, Traffic, Ultra Buy (UB), Wildhorse, 1st Class, 1893], but it was the only ad in our sample for these brands.

For keywords/phrases, ‘naked’ (31.4%), ‘wild’ (26.9%), the phrase ‘tobacco and water’ (18.7%), ‘simple’ (14.8%), and ‘different’ (11.9%) were the most common overall (Supplementary file Table 2). Descriptors like ‘naked’ and ‘wild’ emerged only in 2022, whereas ‘tobacco and water’, ‘simple’, and ‘different’ were more common in the years 2019–2021.

The most prevalent textual references were farming/growing practices (15.6%), environmental protections via consumer actions (10.7%), and environmental events (8.2%). References to farming/growing practices were most common in 2023 (occurring in 25.2% of ads), while references to environmental protection via the consumer were more common in 2019 (19.8%), 2020 (19.7%) and 2021 (17.7%). References to environmental events were most common in 2021 (35.3%).

Imagery of flora (47.8%), great outdoors settings (27.7%), and farming or gardening (18.3%), were most common overall. Flora imagery occurred most commonly in 2022 (appearing in 77.6% of ads), while great outdoors imagery occurred most commonly in 2019 (58.4%), and farming imagery occurred most commonly in 2021 (55.9%).

Promotions related to greenwashing were less common among this sample, with 5.1% of ads containing eco-related sweepstakes, 3.9% containing eco-related giveaways and 1.6% featuring paperless coupons. Ads featuring eco-related sweepstakes were found in all years except 2020, while no instances of eco-related giveaways or paperless coupons were found after 2020.

Brands varied in their use of greenwashing tactics (Supplementary file Table 3). Hestia ads predominantly featured the descriptors ‘naked’ (74.6%) and ‘wild’ (63.9%), and less commonly used the term ‘natural’ (15.6%). Just over 15% of Hestia ads featured textual references to farming or growing practices, while 67.3% featured flora imagery. Natural American Spirit ads used descriptors such as ‘different’ (50.9%), ‘simple’ (41.1%), ‘real’ (33.9%), ‘organic’ (33.0%), ‘earth-friendly’ (23.2%), ‘tobacco and water’ (22.3%), and ‘recyclable’ (22.3%). These ads also used a variety of textual references, including messaging about environmental protections that could be taken by the consumer (38.4%), environmental events (34.8%), and farming or growing practices (32.1%). Over half (58.9%) of Natural American Spirit ads featured flora imagery, while 38.4% featured farming or gardening imagery and 31.3% featured imagery of the great outdoors. Around 17% of Natural American Spirit ads featured eco-related giveaways. Among Winston ads, 45.0% used the term ‘tobacco and water’, while 19.0% used the term ‘plant-based’. Broader textual references were used more sparingly. Winston ads had 47.0% depicting the great outdoors and 26.0% featuring farming or gardening imagery. Marlboro ads relied most heavily on great outdoors imagery (92.7%), eco-related sweepstakes (41.5%), and textual references to environmental protections via the consumer (19.5%) or via charitable activity (12.2%).

## DISCUSSION

Findings from this study indicate that greenwashing is a common tactic in cigarette advertising. Although certain companies have been restricted from using the terms ‘natural’ and ‘additive-free’^15^, cigarette brands are leveraging a diverse range of tactics that could make their products seem eco-friendly, natural, and potentially less harmful. Commonly used tactics included specific phrases such as ‘naked’, ‘wild’, and ‘tobacco and water’; imagery of flora and the great outdoors; and general references to eco-friendly actions such as farming and growing practices. These findings are consistent with prior work documenting the wide range of tactics tobacco companies use to greenwash their products, including the use of flora and depictions of the outdoors, as well as language extending beyond ‘natural’ and ‘additive-free’^6,7,16,17,20^.

Despite differences in the overall marketing and sale strategies, the use of greenwashing appeared concentrated among four brands: Hestia, Natural American Spirit, Winston, and Marlboro. That said, our study identified 12 brands that featured at least one greenwashing tactic in at least one ad. Natural American Spirit’s marketing has received considerable attention and multiple studies have linked its use of greenwashing tactics to inaccurate reduced product risk perceptions^11,13,14^. While research has previously documented Winston’s and Marlboro’s use of greenwashing^5,23^, this study identified a newer cigarette brand, Hestia, that prominently used greenwashing. Greenwashing tactics appeared in a considerable portion of this brand’s advertising. The introduction of this brand appears to partly explain temporal shifts in the language most commonly found in the sample of ads (e.g. emergence of descriptors ‘naked’ and ‘wild’ in 2022 to reference natural tobacco^24^). The use of greenwashing tactics among these brands is concerning given the reach and potential appeal of these brands among young people. Marlboro remains the most popular brand of cigarettes among US youth and adults^25^, and preference for Natural American Spirit cigarettes has also increased over time, particularly for younger adults^26^. Currently, Hestia is not a widely available brand of cigarettes, but given their potential to appeal to young people^27^ and the impact of greenwashing on consumer reduced harm perceptions and potential appeal to youth and young adults^28^, it is worth continuing to monitor this brand.

In the US, tobacco companies are not allowed to advertise their products as presenting reduced risk or exposure without obtaining a modified risk order from the FDA^2^. None of the products in this study has been granted a modified risk order. Despite this, research has found that use of many of the greenwashing tactics identified in this study are associated with inaccurate reduced risk perceptions^11,13,14^. For instance, a recent randomized controlled experiment found that the use of two widely used greenwashing tactics (flora imagery and claims about eco-friendly farming practices), led to inaccurate perceptions of lower product harm, addictiveness, and nicotine content among people who viewed the ads^11^.

Collectively, our findings and those of earlier studies underscore the need for regulatory measures that address greenwashing tactics in cigarette advertising. Prior work has demonstrated that the tobacco industry can quickly pivot to new, but similar, tactics when confronted with restrictions on use of a specific phrase (i.e. relying on the term ‘tobacco and water’ after ‘additive-free’ was restricted)^5^. Thus, a way to address the use of greenwashing tactics in cigarette ads may be to adopt a more comprehensive approach, rather than to simply restrict the use of specific terms. For example, such regulations could include plain packaging requirements or content-neutral approaches such as geographically- or audience-based restrictions on where tobacco advertising can be advertised.

### Limitations

This is a cross-sectional assessment of the presence and type of greenwashing tactics present in cigarette ads in the US from 2019–2023. These data do not directly connect to use patterns or impact of purchasing, so we cannot conclude a causal role of ads on product purchases. While we believe our sample provides a comprehensive inventory of the range of greenwashing tactics used by cigarette brands, we may not have captured all ads that ran in the US during that time period, particularly point-of-sale advertising, which is an important channel for cigarette marketing^29^. While companies typically have consistent themes and branding as part of larger advertising campaigns disseminated in multiple channels^30^, it is possible that point-of-sale advertising contained different greenwashing tactics than identified here. Further, we did not capture money spent on advertising or consumer exposure data, so we are unable to make inferences about the reach of any given tactic. Finally, this study focused only on cigarette advertising. Research has found that greenwashing tactics are being used in other tobacco products^31^; thus, systematic monitoring of greenwashing in other tobacco products could be informative.

## CONCLUSIONS

This study identified a comprehensive inventory of greenwashing tactics currently used by cigarette companies. Greenwashing continues to be used in cigarette advertising, including use of tactics associated with inaccurate modified risk perceptions. If further studies strengthen the evidence, regulations to limit greenwashing in tobacco advertising may be justified.

## Supplementary Material



## Data Availability

The data supporting this research are available from the authors on reasonable request.
