# Supplementary material for: A descriptive content analysis of greenwashing tactics used in US cigarette advertisements between 2019–2023
Source: Tob Prev Cessat. 2026 Apr 30;12:10.18332/tpc/213722. doi: 10.18332/tpc/213722 (PMC13131989; doi:10.18332/tpc/213722)
Supplement: Supplementary file 1 [file TPC-12-22-s1.pdf]

SUPPLEMENTARY FILE

Table 1. Codebook for a descriptive content analysis of greenwashing features in 487 U.S. cigarette advertisements from 2019-2023

Code	Description
**Descriptors**	
Naked	Ad contains the word naked.
Wild	Ad contains the word wild.
Tobacco and Water	Ad contains the phrase tobacco and water or a variation thereof (e.g., "Tobacco. Water.").
Simple	Ad contains the word simple or a variation thereof (e.g., simplicity, simply).
Different	Ad contains the word simple or a variation thereof (e.g., simplicity, simply).
Organic	Ad contains the word organic or a variation thereof (e.g., organically).
Natural	Ad contains the word natural or a variation thereof (e.g., naturally, naturalness).
Real	Ad contains the word real or a variation thereof (e.g., realness).
Earth-friendly	Ad contains the phrase earth-friendly.
Plant-based	Ad contains the word plant-based.
Recyclable	Ad contains the word recyclable or a variation thereof (e.g., recycling, recycle).
Whole leaf	Ad contains the phrase whole leaf.
Litter	Ad contains the word litter or a variation thereof (e.g., littering, anti-litter, anti-littering).
Sustainable	Ad contains the word sustainable or a variation thereof (e.g., sustainability, sustainably).
Plant	Ad contains the word plant.
Green	Ad contains the word green.
Biodegradable	Ad contains the word biodegradable.
Nature	Ad contains the word nature.
Pure	Ad contains the word pure or a variation thereof (e.g., purity, purely).
Environment	Ad contains the word environment or a variation thereof (e.g., environmentally, environmental).
Unadulterated	Ad contains the word unadulterated.
Additive-free	Ad contains the phrase additive-free.
Eco-friendly	Ad contains the word eco-friendly.
Eco-conscious	Ad contains the word eco-conscious.
Trash	Ad contains the word trash.
**Imagery**	
Flora	Ad contains imagery of flowers, plants or leaves.
Great outdoors	Ad features a 'great outdoors' setting.

Farming/gardening	Ad features farming/gardening imagery.
Recycling logo	Ad features the recycling logo.
Programme for the Endorsement of Forest Certification logo	Ad features the Programme for the Endorsement of Forest Certification logo.
**Textual references**	
Farming/growing practices	Ad references how product was grown/harvested or other references to farming as related to the product.
Environmental protection via consumer	Ad references how consumers can take action to protect or care for the environment.
Environmental events	Ad references environmental events or holidays.
Environmental protection via business practices	Ad references how manufacturer/company/brand is protecting or caring for the environment via their business practices.
Environmental protection via charitable activities/partnerships	Ad references how manufacturer/company/brand is protecting or caring for the environment via their charitable activities or partnerships.
Anti-littering	Ad encourages consumers to not litter.
Recycling	Ad encourages consumers to recycle or conveys how product/company is recycling or recyclable.
Energy/water	Ad references company/manufacturer/brand use of clean or reduced energy or water.
**Promotions**	
Eco-related sweepstakes	Ad features a sweepstakes with eco-, nature- or outdoors-related prizes.
Eco-related giveaways	Ad gives away or features an opportunity to receive an eco-, nature or outdoors-related item.
Paperless coupons	Ad features paperless coupons or price promotions/discounts.
Note: Codes were developed based on existing research [6,7,16,20] and on an initial review of advertisements.	

Table 2. Greenwashing tactics by year from 2019-2023 for a descriptive content analysis of 487 U.S. cigarette advertisements

	Year											
	2019 (n=101)		2020 (n=61)		2021 (n=34)		2022 (n=152)		2023 (n=139)		Total	
	n	%	n	%	n	%	n	%	n	%	n	%
**Descriptors**												
Naked *	0	0.0	0	0.0	0	0.0	99	65.1	54	38.9	153	31.4
Wild *	0	0.0	0	0.0	0	0.0	78	51.3	53	38.1	131	26.9
Tobacco and Water *	30	29.7	30	49.2	2	5.9	21	13.8	8	5.8	91	18.7
Simple *	42	41.6	10	16.4	0	0.0	10	6.6	10	7.2	72	14.8
Different *	29	28.7	10	16.4	7	20.6	10	6.6	2	1.4	58	11.9
Organic *	13	12.9	7	11.5	12	35.3	11	7.2	6	4.3	49	10.1
Natural *	0	0.0	1	1.6	2	5.9	19	12.5	24	17.3	46	9.5
Real *	29	28.7	5	8.2	0	0.0	1	0.7	4	2.9	39	8.0
Earth-friendly *	12	11.9	6	9.8	6	17.7	2	1.3	1	0.7	27	5.5
Plant-based *	0	0.0	7	11.5	5	14.7	6	4.0	9	6.5	27	5.5
Recyclable *	13	12.9	11	18.0	0	0.0	0	0.0	1	0.7	25	5.1
Whole leaf *	8	7.9	4	6.6	5	14.7	5	3.3	0	0.0	22	4.5
Litter *	6	5.9	10	16.4	1	2.9	4	2.6	0	0.0	21	4.3
Sustainable *	7	6.9	2	3.3	4	11.8	6	4.0	0	0.0	19	3.9
Plant *	5	5.0	0	0.0	3	8.8	0	0.0	6	4.3	14	2.9
Green *	6	5.9	2	3.3	0	0.0	0	0.0	3	2.2	11	2.3
Biodegradable *	0	0.0	0	0.0	0	0.0	0	0.0	10	7.2	10	2.1
Nature	0	0.0	2	3.3	1	2.9	2	1.3	2	1.4	7	1.4
Pure	1	1.0	0	0.0	0	0.0	3	2.0	1	0.7	5	1.0
Environment	0	0.0	2	3.3	0	0.0	0	0.0	2	1.4	4	0.8
Unadulterated	0	0.0	0	0.0	0	0.0	2	1.3	2	1.4	4	0.8
Additive-free	0	0.0	0	0.0	0	0.0	0	0.0	1	0.7	1	0.2
Eco-friendly	0	0.0	1	1.6	0	0.0	0	0.0	0	0.0	1	0.2

Eco-conscious	0	0.0	1	1.6	0	0.0	0	0.0	0	0.0	1	0.2
Trash	0	0.0	0	0.0	0	0.0	0	0.0	0	0.0	0	0.0
**Imagery**												
Flora *	39	38.6	17	27.9	20	58.8	118	77.6	39	28.1	233	47.8
Great outdoors *	59	58.4	23	37.7	4	11.8	23	15.1	26	18.7	135	27.7
Farming/gardening *	12	11.9	6	9.8	19	55.9	29	19.1	23	16.6	89	18.3
Recycling logo *	9	8.9	5	8.2	2	5.9	0	0.0	1	0.7	17	3.5
Programme for the Endorsement of Forest Certification logo	3	3.0	1	1.6	0	0.0	0	0.0	0	0.0	4	0.8
**Textual references**												
Farming/growing practices *	14	13.9	6	9.8	8	23.5	13	8.6	35	25.2	76	15.6
Environmental protection via consumer *	20	19.8	12	19.7	6	17.7	11	7.2	3	2.2	52	10.7
Environmental events *	7	6.9	10	16.4	12	35.3	7	4.6	4	2.9	40	8.2
Environmental protection via business practices *	17	16.8	3	4.9	7	20.6	9	5.9	1	0.7	37	7.6
Environmental protection via charitable activities/partnerships *	8	7.9	4	6.6	14	41.2	4	2.6	1	0.7	31	6.4
Anti-littering *	14	13.9	7	11.5	3	8.8	6	4.0	0	0.0	30	6.2
Recycling *	2	2.0	9	14.8	0	0.0	1	0.7	1	0.7	13	2.7
Energy/water *	0	0.0	0	0.0	6	17.7	0	0.0	3	2.2	9	1.9
**Promotions**												
Eco-related sweepstakes *	17	16.8	0	0.0	1	2.9	3	2.0	4	2.9	25	5.1
Eco-related giveaways *	15	14.9	4	6.6	0	0.0	0	0.0	0	0.0	19	3.9
Paperless coupons *	5	5.0	3	4.9	0	0.0	0	0.0	0	0.0	8	1.6

*Denotes chi-square test of prevalence of tactics across years significant at $p<.05$

Table 3. Greenwashing tactics by brand for a descriptive content analysis of 487 U.S. cigarette advertisements from 2019-2023

	Brand														Total	
	Hestia (n=205)		Natural American Spirit (NAS) (n=112)		Winston (n=100)		Marlboro (n=41)		Nat Sherman (n=12)		Aura (n=7)		Other^ (Parliament, Camel, Leaf by Lane, Lucky Strike, Signal, Very Low Nicotine (VLN), etc.) (n=10)			
	n	%	n	%	n	%	n	%	n	%	n	%	n	%	n	%
**Descriptors**																
Naked *	153	74.6	0	0.0	0	0.0	0	0.0	0	0.0	0	0.0	0	0.0	153	31.4
Wild *	131	63.9	0	0.0	0	0.0	0	0.0	0	0.0	0	0.0	0	0.0	131	26.9
Tobacco and Water *	1	0.5	25	22.3	45	45.0	0	0.0	12	100.0	7	100.0	1	10.0	91	18.7
Simple *	0	0.0	46	41.1	11	11.0	2	4.9	12	100.0	0	0.0	1	10.0	72	14.8
Different *	0	0.0	57	50.9	0	0.0	1	2.4	0	0.0	0	0.0	0	0.0	58	11.9
Organic *	12	5.9	37	33.0	0	0.0	0	0.0	0	0.0	0	0.0	0	0.0	49	10.1
Natural *	32	15.6	2	1.8	10	10.0	0	0.0	0	0.0	1	14.3	1	10.0	46	9.4
Real *	1	0.5	38	33.9	0	0.0	0	0.0	0	0.0	0	0.0	0	0.0	39	8.0
Earth-friendly *	1	0.5	26	23.2	0	0.0	0	0.0	0	0.0	0	0.0	0	0.0	27	5.5
Plant-based *	8	3.9	0	0.0	19	19.0	0	0.0	0	0.0	0	0.0	0	0.0	27	5.5
Recyclable *	0	0.0	25	22.3	0	0.0	0	0.0	0	0.0	0	0.0	0	0.0	25	5.1
Whole-leaf *	0	0.0	19	17.0	3	3.0	0	0.0	0	0.0	0	0.0	0	0.0	22	4.5
Litter *	0	0.0	20	17.9	0	0.0	1	2.4	0	0.0	0	0.0	0	0.0	21	4.3
Sustainable *	0	0.0	19	17.0	0	0.0	0	0.0	0	0.0	0	0.0	0	0.0	19	3.9
Plant	6	2.9	8	7.1	0	0.0	0	0.0	0	0.0	0	0.0	0	0.0	14	2.9
Green	3	1.5	6	5.4	1	1.0	0	0.0	1	8.3	0	0.0	0	0.0	11	2.3
Biodegradable *	10	4.9	0	0.0	0	0.0	0	0.0	0	0.0	0	0.0	0	0.0	10	2.1
Nature *	2	1.0	4	3.6	0	0.0	0	0.0	0	0.0	1	14.3	0	0.0	7	1.4

Pure	3	1.5	0	0.0	1	1.0	0	0.0	0	0.0	0	0.0	1	10.0	5	1.0
Environment *	2	1.0	1	0.9	0	0.0	0	0.0	0	0.0	1	14.3	0	0.0	4	0.8
Unadulterated	4	2.0	0	0.0	0	0.0	0	0.0	0	0.0	0	0.0	0	0.0	4	0.8
Additive-free	1	0.5	0	0.0	0	0.0	0	0.0	0	0.0	0	0.0	0	0.0	1	0.2
Eco-friendly	0	0.0	1	0.9	0	0.0	0	0.0	0	0.0	0	0.0	0	0.0	1	0.2
Eco-conscious *	0	0.0	0	0.0	0	0.0	0	0.0	0	0.0	1	14.3	0	0.0	1	0.2
Trash	0	0.0	0	0.0	0	0.0	0	0.0	0	0.0	0	0.0	0	0.0	0	0.0
**Textual references**																
Farming/growing practices *	31	15.1	36	32.1	8	8.0	0	0.0	0	0.0	0	0.0	1	10.0	76	15.6
Environmental protection via consumer *	0	0.0	43	38.4	0	0.0	8	19.5	0	0.0	1	14.3	0	0.0	52	10.7
Environmental events *	0	0.0	39	34.8	0	0.0	0	0.0	0	0.0	1	14.3	0	0.0	40	8.2
Environmental protection via business practices *	3	1.5	29	25.9	0	0.0	4	9.8	0	0.0	1	14.3	0	0.0	37	7.6
Environmental protection via charitable activities/partnerships *	0	0.0	26	23.2	0	0.0	5	12.2	0	0.0	0	0.0	0	0.0	31	6.4
Anti-littering *	0	0.0	30	26.8	0	0.0	0	0.0	0	0.0	0	0.0	0	0.0	30	6.2
Recycling *	0	0.0	12	10.7	0	0.0	0	0.0	0	0.0	1	14.3	0	0.0	13	2.7
Energy/water *	0	0.0	8	7.1	0	0.0	1	2.4	0	0.0	0	0.0	0	0.0	9	1.9
**Imagery**																
Flora *	138	67.3	66	58.9	12	12.0	4	9.8	9	75.0	2	28.6	2	20.0	233	47.8
Great outdoors settings *	6	2.9	35	31.3	47	47.0	38	92.7	0	0.0	4	57.1	5	50.0	135	27.7
Farming/gardening *	17	8.3	43	38.4	26	26.0	1	2.4	0	0.0	0	0.0	2	20.0	89	18.3
Recycling logo *	0	0.0	16	14.3	0	0.0	0	0.0	0	0.0	1	14.3	0	0.0	17	3.5
Programme for the Endorsement of Forest Certification logo *	0	0.0	0	0.0	0	0.0	4	9.8	0	0.0	0	0.0	0	0.0	4	0.8
**Promotions**																

Eco-related sweepstakes *	0	0.0	3	2.7	5	5.0	17	41.5	0	0.0	0	0.0	0	0.0	25	5.1
Eco-related giveaways *	0	0.0	19	17.0	0	0.0	0	0.0	0	0.0	0	0.0	0	0.0	19	3.9
Paperless coupons *	0	0.0	8	7.1	0	0.0	0	0.0	0	0.0	0	0.0	0	0.0	8	1.6

^ 1 ad contained 8 Premier Manufacturing cigarette brands advertised (Ace, Manitou, Shield, Traffic, Ultra Buy (UB), Wildhorse, 1st Class, 1893), but it was the only ad in our sample for these brands.

*Denotes chi-square test of prevalence of tactics across years significant at *p*<.05

© 2026 Ibrahim M. et al.
